# A Concise Review of MicroRNA-383: Exploring the Insights of Its Function in Tumorigenesis

**DOI:** 10.7150/jca.64846

**Published:** 2022-01-01

**Authors:** Qian Yi, Wei Xie, Wei Sun, Weichao Sun, Yi Liao

**Affiliations:** 1 The Central Laboratory, Shenzhen Second People's Hospital/First Affiliated Hospital of Shenzhen University Health Science Center, Shenzhen, Guangdong 518035, P.R. China.; 2 Department of Physiology, School of Basic Medical Science, Southwest Medical University, Luzhou, Sichuan province 646099, P.R. China.; 3 Department of Orthopedics, Shenzhen Second People's Hospital/First Affiliated Hospital of Shenzhen University Health Science Center, Shenzhen, Guangdong 518035, P.R. China.; 4 Laboratory of Anesthesia and Organ Protection, Southwest Medical University, Luzhou, Sichuan province 646099, P.R. China.

**Keywords:** miR-383, Cancer, Proliferation, Apoptosis, Invasion, Metastasis

## Abstract

MicroRNAs (miRNAs) are small noncoding RNAs that commonly have 18-22 nucleotides and play important roles in the regulation of gene expression via directly binding to the 3'-UTR of target mRNAs. Approximately 50% of human genes are regulated by miRNAs and they are involved in many human diseases, including various types of cancers. Recently, microRNA-383 (miR-383) has been identified as being aberrantly expressed in multiple cancers, such as malignant melanoma, colorectal cancer, hepatocellular cancer, and glioma. Increasing evidence suggests that miR-383 participates in tumorigenic events including proliferation, apoptosis, invasion, and metastasis as well as drug resistance. Although downstream targets including *CCND1, LDHA, VEGF,* and *IGF* are illustrated to be regulated by miR-383, its roles in carcinogenesis are still ambiguous and the underlying mechanisms are still unclear. Herein, we review the latest studies on miR-383 and summarize its functions in human cancers and other diseases. The goal of this review is to provide new strategies for targeted therapy and further investigations.

## 1. Introduction

Cancer is one of the most life-threatening diseases and the leading cause of death in humans 1,2. Lung cancer is the most commonly diagnosed cancer and the leading cause of cancer-related death among males, while breast cancer is the most prevalent among females 3. There are many risk factors that contribute to cancer development, such as gender, age, region and race. The occurrence, development, and pathogenesis of different types of cancer can vary widely. Therefore, investigating the specific molecular mechanisms of different types of cancer may provide us with new ideas to develop novel and effective therapies for cancer treatment.

MicroRNAs (miRNAs) are characterized as a class of small noncoding RNAs comprised of approximately 18-22 nucleotides 4. The critical role of miRNAs is to regulate gene expression in a post-transcriptional manner. Through binding to the specific sites of the 3'-UTRs of targeted mRNAs, miRNAs mediate their degradation and translational repression. Approximately 50% of human genes are regulated by miRNAs, including tumor suppressor genes and oncogenes 5, 6. Therefore, miRNAs play important regulatory roles in multiple biological progressions, such as cell proliferation, apoptosis, and carcinogenesis. Meanwhile, studies have also found that the expression of miRNAs is differ among the early, middle, and advanced stages of various cancers, thus indicating that miRNAs could be clinical or diagnostic biomarkers. Exploring the role and mechanism of miRNAs in tumors is critical for the development of unique and effective miRNA-based therapies.

MicroRNA-383 (miR-383) is located on chromosome 8p22, within the third intron of the sarcoglycan zeta (SGCZ) gene 7. It has been reported that chromosome 8p is frequently lost or mutated and the loss of chromosome 8p is a characteristic of prostate cancer, with more than half of the loss being due to genomic alterations 8, 9. The genomic alterations on chromosome 8p21-p23 also frequently occur in stage I squamous cell lung carcinoma 10. Therefore, the expression of miR-383 was found to be aberrant in multiple types of cancer. In lung cancer, the expression of miR-383 was markedly lower than in the non-cancerous lung tissues, and further reduced in advanced-stage carcinomas 11. Down-regulation of miR-383 was also observed in cancers that include glioma, hepatocellular cancer, and breast cancer **(Table 1).** However, in primary human malignant melanoma, the expression level of miR-383 was higher than normal epidermal melanocytes 12. Upregulated expression and oncogenic function of miR-383 has also observed in cholangiocarcinoma, epithelial ovarian cancer, and renal cell carcinoma. These results suggest that miR-383 might play significant roles in tumor progression and its functions in tumorigenesis are controversial.

In this paper, we review the roles and mechanisms of miR-383 in cancer cell proliferation, apoptosis, invasion and metastasis, development and differentiation, as well as its functions in other human diseases. This review aims to provide evidence or suggestions for further investigations and clinical applications regarding miR-383.

## 2. miR-383 in cell proliferation

Several studies have shown that miR-383 mediates cell proliferation via regulating the expression of proliferation-associated genes. Cyclin D1 is well-known in regulating cell proliferation and has been demonstrated to be overexpressed in many human cancers 60. It has been reported that CREPT can bind to the promoter of Cyclin D1, enhancing its transcription and expression 61. Li *et al*. illustrated that miR-383 directly binds to the 3'-UTR of CREPT mRNA and inhibits the expression of CREPT and cyclin D1, thereby suppressing cell growth and colony formation of colorectal cancer cells 28. It has been demonstrated that miR-383 can directly bind to Cyclin D1 mRNA, inducing cell cycle arrest at the G0/G1 phase and inhibiting glioma cell growth 21.

A proliferating-inducing ligand (APRIL) belongs to the tumor necrosis factor super-family. As a cytokine, it stimulates cell proliferation and modulates cell apoptosis, playing important roles in tumorigenesis 62, 63. Overexpression of miR-383 in colon cancer cells was associated with the decreased expression of APRIL and the inhibition of cell proliferation 48. The direct binding between miR-383 and APRIL mRNA was recently identified in hepatocellular carcinoma cells. Overexpression of miR-383 also induced cell cycle arrest in G0/G1 phase and inhibited the proliferation of HepG2 and SK-Hep-1 cells 30.

The expression of miR-383 was also decreased in medulloblastoma, ovarian cancer, pancreatic carcinoma, and colorectal cancer **(Table 1).** In medulloblastoma, miR-383 regulated the transcription and translation of *PRDX3*. Ectopic expression of miR-383 significantly suppressed cell growth 34. In ovarian cancer, miR-383-5p suppressed cell proliferation *in vitro* and inhibited tumor growth *in vivo* by targeting *TRIM27* 26. Overexpression of miR-383 markedly suppressed the proliferation of SW620 and HCT116 human colorectal cancer cells through targeting *PAX6* 27. Moreover, miR-383 inhibited the gastric mucosa-associated lymphoid tissue lymphoma proliferation via targeting *ZEB2* 50. In pancreatic carcinoma, miR-383 inhibited the expression of *ROBO3* and suppressed cell growth 37. In testicular embryonal carcinoma, miR-383 targeted the tumor suppressor *IRF1* to reduce the expression of Cyclin D1, CDK2, and p21, which inhibited proliferation through inactivation of the pRb pathway 23, 64. The proliferation-regulation role of the miR-383-LDHA axis has also been demonstrated in hepatocellular cancer cells such as HepG2 and SMMC-7721 cells 29. Additionally, miR-383-5p suppresses cell proliferation via directly targeting *CIP2A* in lung adenocarcinoma 16. Furthermore, miR-383 targeted *VEGF* to suppress glioma-exposed endothelial cells proliferation 20. In esophageal squamous carcinoma cells, miR-383 decreased the expression of 5S rRNA and intensified the rpL11-c-Myc interaction, resulting in the attenuation of c-Myc and inhibition of cell proliferation 39. In testicular embryonal carcinoma cells, miR-383 impaired the phosphorylation of H2AX and induced cell cycle arrest via the direct targeting of *PNUTS* mRNA 22. The anti-proliferation function of miR-383 has also been reported in other types of cancer; its expression in different cancers is summarized in **Table 1**.

**Figure 1:**
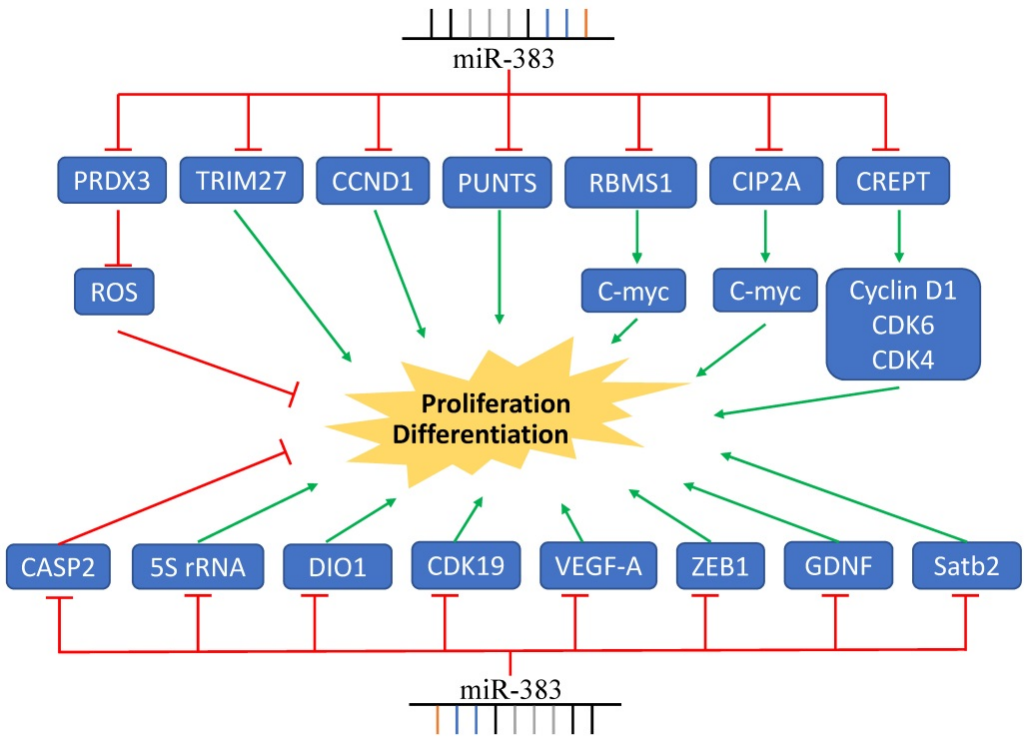
MiR-383 Regulates Cancer Cell Proliferation. MiR-383 overexpression suppressed cell proliferation via inhibition of the expression of *CCND1*, *CREPT, VEGF-A*, and others, while it increased proliferation by inhibiting the expression of *CASP2*. Green arrows indicate promotion and red lines indicate suppression.

The expression of miR-383 was found to be frequently decreased in various malignant tumors; however, it was upregulated in cholangiocarcinoma tissues and acted as an oncogenic miRNA by inhibiting the expression of tumor suppressor gene *IRF1* 54. Sheng *et al.* also reported that the expression of miR-383 was significantly elevated in immortal human epithelial ovarian cancer cell lines and human epithelial ovarian cancer tumors. They found that miR-383 targeted *CASP2*, and stable knockdown of miR-383 expression was associated with the suppression of cell proliferation 55. The upregulation of miR-383 was also found in clear cell renal cell carcinoma. MiR-383 was negatively associated with the expression of *DIO1*, which was reported to inhibit the proliferation of renal cancer cells 59. Together, these data indicate that the role of miR-383 in tumor proliferation is critical, complex, and involves multiple signaling pathways **(Figure 1)**.

## 3. miR-383 in cell apoptosis

A large body of evidence has uncovered the relationship between impaired cell apoptosis and cancer development. Bcl-2 family signaling consists of Bax pro-apoptosis protein, Bcl-2 anti-apoptosis protein, and BH3-only protein. It is a classical mitochondria pathway that mediates cell apoptosis. Recently, miR-383 has been found to promote medulloblastoma cell apoptosis via repressing the expression of *PRDX3*, up-regulating cleaved PARP expression, and reducing the expression of BCL-XL and/or BCL-2 34. Moreover, in human retinal pigment epithelial cells, a high glucose treatment could increase the expression of miR-383, promote reactive oxygen species (ROS) formation, downregulate Bcl-2 and Bax expression, and induces apoptosis by repressing *PRDX3* 65.

Ultraviolet radiation (UV) or ionizing radiation can induce DNA damage and activate ATR and ATM, members of the phosphatidylinositol 3-kinase-related kinase (PIKK) family 66. Studies have shown that ATR plays a significant role in DNA damage response, proliferation, and apoptosis 55,56. In A431 melanoma cells, Stat3 decreased the expression of miR-383 and, as the direct target of miR-383, ATR expression was increased. MiR-383 also mediated ATR activity to control DNA damage and affect cell apoptosis 67. Studies have also reported that ATR was a target for miR-383 in canine malignant melanoma, indicating that miR-383 may be involved in melanoma tumorigenesis by inhibiting DNA repair or apoptosis 68.

Gadd45a, Gadd45b, and Gadd45g constitute the Gadd45 family. Via interactions with PCNA, p21, and cdc2/Cyclin B1, they regulate cell proliferation, the cell cycle, and apoptosis 69. Recently, Gadd45g was found as a direct target of miR-383. MiR-383 promoted apoptosis and increased the sensitive of breast cancer cells to both UV irradiation and cisplatin treatment 42. The caspase family are key mediators in the maintenance of cell homeostasis by regulating inflammatory response and apoptosis. The expression of miR-383 was positively related to the number of apoptotic nuclei in brain infarct area of ischemic stroke in a rat model. The expression of cleaved caspase-3 and cleaved PARP were also found to increase after ischemic stroke. These results shown us that miR-383 may promote apoptosis in ischemic stroke 70. In non-small cell lung cancer, overexpression of miR-383 induced apoptosis via targeting the Wnt/β-catenin signaling pathway 13. In human glioma cells, miR-383 overexpression increases the rate of apoptotis of U251 and U87 cells from 8.0% and 1.9% to 36.6% and 16.9%, respectively 19.

The above studies show that, while controversial, there are apoptosis-promoting functions of miR-383. Shuai *et al*. found that propofol treatment significantly decreased the expression of miR-383. The reduced expression of Bcl-2 and increased expression of Bax induced by propofol was inhibited by miR-383 mimic treatment. These data indicate that miR-383 inhibited the neuron apoptosis by regulating the expression of Bcl-2 and Bax 71. Moreover, Resveratrol treatment reduced the expression of miR-383-5p in human podocytes, and it effectively inhibited high-glucose-induced apoptosis via stimulating autophagy 72. Furthermore, in human epithelial ovarian cell lines, the overexpression of miR-383 decreased the expression of caspase-2, indicating that miR-383 was acting as an oncogene in human epithelial ovarian cell lines 55. Furthermore, in homocysteine-induced endothelial injury in rat coronary arteries, miR-383-3p negatively regulated the expression of IL1R2 and caspase-1. Therefore, miR-383-3p may function by decreasing cell apoptosis of coronary artery endothelial cells 73.

## 4. miR-383 in cancer invasion and metastasis

Cancer invasion and metastasis is a complex process that is the major obstacle to cancer treatment. The epithelial-to-mesenchymal transition (EMT) is a process characterized by a decrease in E-cadherin expression and increase of N-cadherin/Vimentin expression, which confers cells migratory and invasive properties. Recently, it has been reported that miR-383 repressed the metastasis of pancreatic carcinoma through regulating EMT. MiR-383 decreased *ROBO3* expression and inhibited the Wnt/β-catenin signaling pathway, resulting in an increased expression of E-cadherin and decreased expression of Vimentin/N-cadherin 37. In nasopharyngeal carcinoma, miR-383-3p suppressed *HMGA2* expression and inhibited the invasion of NPC cells via modulating the EMT process 53.

Lactate dehydrogenase A (LDHA) is an important enzyme involved in the regulation of the glycolysis pathway and cell metabolism. Recently, its critical roles in cell proliferation, glycolysis, and invasion of cancer cells have been reported. LDHA is a direct target of miR-383, with the enhanced invasive capacity of HepG2 and SMMC-7721 cells induced by LDHA overexpression being abolished by miR-383 overexpression 29. In Li and colleagues' study, SKOV3 ovarian cells were transfected with a miR-383 inhibitor and OVCAR3 ovarian cells was transfected with miR-383 mimics. These two cell lines have relatively high and low miR-383 expression, respectively. The transwell assays showed that the invasive ability of OVCAR3 and SKOV3 cells were decreased and increased after transfection, respectively. The authors found that the function of miR-383 in suppressing ovarian cancer cell invasion was mediated by LDHA 25.

IGF1R signaling is constitutively active in many human cancers and the IGF1R/AKT/MMP2 axis plays crucial roles in tumor invasion. In human glioma cancer cells, miR-383 expression was suppressed. MiR-383 mimic significantly reduced the invasive ability of U87MG glioma cell, while miR-383 suppression dramatically increased the A172 cell invasion. Further studies demonstrated that IGF1R was a direct target of miR-383, which regulated the IGF1R/AKT signaling pathway and MMP2 expression, thereby influencing glioma cell invasion 18. Moreover, PARP2 expression was higher in the cervical cancerous tissue compared to the paracancerous tissues. The high expression of PARP2 was associated with high expression of PI3K, AKT, and mTOR. MiR-383 suppressed the expression of PARP2, reduced the activity of PI3K-AKT-mTOR signaling, and inhibited cell migration and invasion 47.

Angiogenesis plays crucial role in cancer cell metastasis, invasion, and tumor progression. Vascular endothelial growth factor (VEGF), an endothelial cell-specific mitogen, is an important mediator of angiogenesis. It has been reported that VEGF was a target gene of miR-383. In glioma-exposed endothelial cells (GECs), miR-383 overexpression decreased the expression of p-VEGFR2, p-FAK, and p-Src mediated by VEGF and inhibited the migration of GECs 20. In human lung cancer cells, miR-383, via inhibiting the expression of EPAS1, repressed the wound healing capacity and invasive capacity of lung cancer cells 15. Moreover, overexpression of miR-383 in SW620 and HCT116 colorectal cells evidently decreased the cell invasion through directly targeting *PAX6* 27. Transfecting HT-29 and LoVo cells with rno-miR-383 mimics significantly reduced the migratory and invasive capacity of colon cancer cells 48. Additionally, in prostate cancer, miR-383 had a strong inhibitory effect on prostate cancer metastasis, mediated by CD44 49.

In contrast, in a study on sorafenib's effects on lung metastasis in hepatocellular carcinoma, researchers found that the expression of miR-383 was up-regulated in lung metastatic tissue, providing new evidence regarding the role of miR-383 in metastasis 57. It has been found that miR-383 inhibition significant repressed human epithelial ovarian cancer cell invasion through the regulation of the caspase-2 gene 55. Interferon regulatory factor 1 (IRF1) is a tumor suppressor in cholangiocarcinoma and was reported to be a direct target of miR-383. High expression of miR-383 induced cholangiocarcinoma cell migration and invasion through repressing the expression of *IRF1* 54.

**Figure 2:**
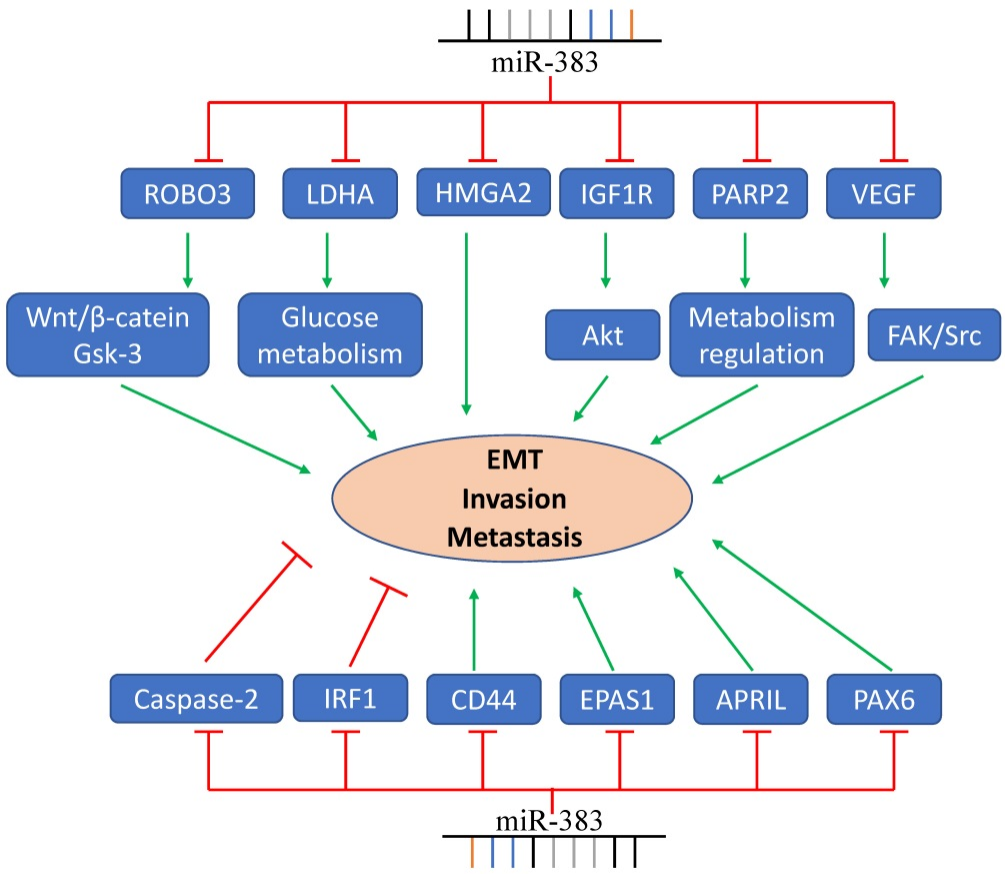
MiR-383 functions in cancer cell EMT, invasion, and metastasis. *LDHA*, *ROBO3*, *PARP2*, and other genes are regulated by miR-383 resulting in the suppression of cancer cell invasion and metastasis. MiR-383 also regulates the expression of IRF- and promotes the cell invasion and metastasis.

These studies show that miR-383 has vital functions in tumor invasion, metastasis, and EMT **(Figure 2)**. Up-regulating the expression of miR-383 inhibits cancer cell invasion and metastasis, while some groups also reported contrary results. Therefore, more studies about the targets and signaling pathway related to miR-383 should be investigated to elucidate the function of miR-383 on cancer cell invasion and metastasis.

## 5. miR-383 in development and cell differentiation

Emerging evidence indicates that microRNAs have important roles in the regulation of osteoblastic differentiation. Recently, miR-383 has been reported as a critical regulator of osteoblastic differentiation. The expression of miR-383 was significantly decreased in the osteoblastic differentiation process of bone marrow mesenchymal stem cells. MiR-383 decreased the expression of alkaline phosphatase, *RUNX2*, and *OCN*, and also suppressed matrix mineralization. *STAB2* has been identified as a direct target of miR-383 in osteoblastic differentiation, with evidence suggesting that the inhibitory role of miR-383 in osteoblastic differentiation may be mediate by *STAB2* 74.

It has been reported that miR-383 influences the characteristics of bone-marrow-derived mesenchymal stem cells and reduce their use in spinal cord injury. Guo *et al*. found that miR-383 targeted binding to the mRNA of *GDNF*, an identified neural growth and survival factor, which inhibited its translation in mesenchymal stem cells (MSCs). The depletion of miR-383 in MSCs increased the expression of GDNF and the therapeutic potentials of MSCs in the treatment of spinal cord injury in a rat model 75. The authors also reported that the expression of vascular endothelial growth factor A (VEGF-A) and cyclin-dependent kinase 19 (CDK-19) were also inhibited by miR-383. MiR-383 suppression increased the proliferation of MSCs and MSC-mediated angiogenesis due to the increased expression of CDK-19 and VEGF-A, respectively. The up-regulated expression of CDK19 and VEGF-A further improved the therapeutic potential of MSCs in treating spinal cord injury (SCI) in rats 76.

MiR-383 has been found to have essential functions in spermatogenesis. It has been reported that the expression of miR-383 in primary spermatocyte was higher than in spermatid, and miR-383 expression was decreased in patients with non-obstructive azoospermia 64. In fragile X mental retardation protein (FMRP) knockout mice testes tissue and *FMRP* downregulated maturation arrest (MA) in patients' testes tissue. The expression of miR-383 was decreased and associated with impaired expression of CDK4 and increased DNA damage 77. The expression of miR-383 was increased during male germ line development, while during female germ line development, it showed a slightly increase but then decreased to a low level. MiR-383 may, through downregulating the expression of cDNMT3B, regulate germ line development in meiotic stages 78.

MiR-383 has also been reported to participate in ovarian follicular and luteal development 79. The expression of miR-383 was significantly downregulated in TGF-β1-treated mouse ovarian granulosa cells 80. Overexpression of miR-383 in ovarian granulosa cells resulted in the decreased expression of c-Myc and increased release of estradiol via targeting of RBMS1 expression 81. Sun *et al*. found that miR-383 upregulated and transactivated miR-320, which regulated the function of granulosa cell by targeting E2F1 and SF-1 82. Although many studies report the specific roles of miR-383 in regulating ovarian follicle development, some research groups have found controversial results. Donadeu and Schauer aspirated the follicular fluid from dominant follicles during the ovulatory and anovulatory seasons to analyze the physiological roles of miRNAs during follicular development; however, they could not detect the expression of miR-383 in follicular fluid 83. In a study to compare the different expressions of miRNAs in follicular fluid from dominant ovulatory, largest subordinate, and dominant anovulatory follicles, the expression of miR-383 was too low to be accurately measured 84. In the early luteal phase of the bovine estrous cycle, the expression of miR-383 was different between granulosa cells of subordinate (SF) and dominant follicles (DF). The SFs expressed abundant miR-383, while it was not detected in granulosa cells of the DFs 85.

## 6. miR-383 in other diseases

Insulin resistance and insufficient pancreatic beta cell insulin secretion are the mainly characteristics of type 2 diabetes. Valeria *et al*. found that the expression of miR-383 was decreased in the islets of adult db/db diabetic mice and high-fat-diet-fed mice 86. Insulin resistance is caused by the disruption of insulin signal transduction, which requires the participation of various proteins, such as insulin, insulin receptor, PI3-K, and glucose transporters. Studies have reported that miR-383 regulated the activity of IGF-1 and IGF-1R, and stimulated the AKT signaling pathway 18, 87. In studies regarding age-associated beta cell dysfunction, researchers have found that miR-383 expression was increased in the islets of older rats. Interestingly, expression changes of miR-383 have no effect on the insulin content, insulin secretion, cell proliferation, and apoptosis 88. In addition, Xia *et al*. reported that free fatty acids can increase miR-383 expression, highlighted by the observation that the expression of DIO1 was inversely associated with miR-383 expression. The authors suggested that miR-383 may influence the different propensities to diet-induced obesity by regulating the DIO1 89.

Stroke is the second leading cause of death in populations over the age of 60 worldwide and neuroinflammation is a main cause of it, with PPARγ playing a beneficial role in ischemia brain injury in stroke 90. In a rat model of middle cerebral artery occlusion, Pei *et al*. found that the expression of PPARγ can be upregulated due to the downregulation of miR-383. These results demonstrated that miR-383 plays an important function in ameliorating injury after focal cerebral ischemia 91. MiR-383 was increased in rats after acute ischemic stroke, and erythropoietin and cyclosporine can decrease miR-383 expression by reducing brain infarct area, indicating that miR-383 participates in the regulation of apoptosis in ischemic stroke 70. Upregulated expression of miR-383 was also reported in a study of ischemic infarction in whole blood 92. However, in the study of the role of miR-383-3p in coronary atherosclerosis, Lian and colleagues reported that miR-383-3p directly targeted IL1R2, demonstrating an anti-inflammatory effect against homocysteine-induced endothelial injury in rat coronary arteries. Their research indicated that miR-383-3p was helpful in preventing coronary atherosclerosis and other cardiovascular diseases 73.

MiRNAs also have essential regulatory roles in the central nervous system. It has been reported that the expression of miR-383 was higher in the marginal division (MrD) than in the hippocampus of Sprague-Dawley rats' brain 93, suggesting miR-383 plays an important role in the learning and memory function of MrD. In the propofol anesthesia-induced cognitive impairment rat model, downregulated miR-383 expression was associated with neuron apoptosis, an increased Bax/Bcl-2 ratio, and decreased expression of PSD95 and CREB. These results suggest that miR-383 protected against hippocampal neuron apoptosis and cognitive impairment 71. The expression of miR-383 in the hypothalamus of leptin-deficient (or non-functional leptin receptor) mice was significantly higher than control C57BL/6 mice. These results indicate that miR-383 expression is modulated by leptin in the hypothalamus and may, via regulating the *POMC* gene, influence the central control energy homeostasis 94.

It has been reported that miR-383 also correlated with various inflammatory diseases. The expression of miR-383 was significantly lower in TNF-α-treated jurkat cells and T cells from rheumatoid arthritis patients 95. In lipopolysaccharide (LPS)-induced RAW264.7 cells, the expression of miR-383 was upregulated, indicating that miR-383 may participate in the regulation of the immune response 96. MiR-383 expression was higher in the additional colonic mucosal tissue of ulcerative colitis (UC) and Crohn disease (CD) patients compared to the control, and CD expressed more miR-383 compared with UC. The differential expression of miR-383 between UC and CD indicates that miR-383 may have important functions in regulating idiopathic inflammatory bowel disease 97.

In some diseases, the expression of miR-338 was downregulated. For example, the expression of miR-383-5p was significantly downregulated in db/db mice and human podocytes after resveratrol treatment and overexpression of miR-383-5p inhibited resveratrol-induced autophagy and apoptosis 72. The expression of miR-383-5p was decreased in rat serum and liver tissue samples kept at 4°C for 12 h, and it may regulate the metabolic pathway, which responds to cold stress 98. Dengue fever (DF) patients had a higher miR-383 expression compared to DF patients with clinical fluid accumulation. These findings suggest that the downregulation of miR-383 could be involved in the complications of DF patients 99. Rats suffering from chronic unpredictable mild stress have upregulated expression of miR-383-5p, while the expression of miR-383 was significantly decreased after electro-acupuncture intervention 100. Therefore, miR-383-5p might affect depression by regulating neurotrophy and neurons apoptosis. However, the expression of miR-383 was upregulated in other diseases. This includes the villi of recurrent pregnancy loss patients, which had increases in miRNA-383, suggesting that miR-383 may regulate the pathogenesis of recurrent pregnancy loss by targeting MALAT1 101. Moreover, miRNA-383 was significantly increased in vitiligo patients. Through regulating *EDN1*, *TYRP1*, and *PRDX3* expression, miRNA-383 participates in the pathogenesis and progression of oxidative stress, autoimmunity, or ER-stress-mediated vitiligo 102.

## 7. Regulation of miR-383 Expression

The expression and function of miRNAs could be regulated by transcriptional regulation and epigenetic modification. In the human genome, miR-383 is locates in the chr8p22 region within the third intron of the SGCZ gene. It has been reported that the loss of heterozygosity at the chr8p22 locus leads to the downregulated expression of miR-383 in prostate cancer 49. DNA methylation was closely related to gene silencing and methyltransferases played essential function in this process. Zhang *et al*. reported that a liver-specific knockout of histone methyltransferase G9a significantly increased miR-383 expression 103. Sun and colleagues reported that the expression of miR-383 was decreased by TGF-β1 in mouse ovarian granulosa cells 80, and they further illustrated that miR-383 was transcriptionally regulated by transcription factor steroidogenic factor-1 (SF-2) 81. Moreover, the transcription activity of miR-383 has also been reported to be downregulated by signal transducer and activator of transcription 3 (STAT3) in human skin cancer 67. Furthermore, it has been reported that HIF-1α promoted macrophage necroptosis by downregulating miR-383 104. In addition, the expression of miR-383 could be regulated by some anti-cancer agents. Lv *et al*. demonstrated that allicin treatment increased the expression of miR-383 in gastric carcinoma 46, while Huang *et al*. reported that resveratrol treatment decreased the expression of miR-383 in podocytes 72. Guo *et al*. also revealed that piperine could decrease miR-383 expression and inhibit proptosis in myocardial ischaemia/reperfusion injury 105.

Interestingly, competing endogenous RNAs (ceRNAs), including long non-coding RNAs (lncRNAs) and circular RNAs (circRNAs), could also regulate the expression and function of miR-383 through influencing the interaction of miR-383 with its target transcript. For example, lncRNA-FGD5-AS1 via sponging miR-383 accelerates the malignant characteristics of esophageal squamous cell carcinoma 38. LncRNA-TMPO-AS1 promotes tumor growth, cell migration, and invasion in pancreatic carcinoma by regulating the miR-383/SOX11 axis 35. Additionally, circRNA-CCS, via sponging miR-383, promotes lung cancer cell growth, metastasis, and predicted poor prognosis 14. Circ-0136666 was reported to facilitate the carcinogenesis of colorectal cancer via targeting the miR-383/CREB1 axis 106. We summarize the ceRNAs-miR-383 networks in **Table 2**.

## 8. Conclusion

In this review, we summarized the roles of miR-383 in human diseases, which may be beneficial for further clinical applications. First, miR-383 has been reported to be dysregulated in various cancers in some studies, whereas it may present a different expression level in an identical cancer in other studies. For instance, miR-383 expression has been reported as decreased in hepatocellular cancer, whereas it was increased in two studies regarding the same type of cancer. Similarly, miR-383 was downregulated in ovarian cancer, while it was significantly upregulated in epithelial ovarian cancer. The possible explanation of the different miR-383 expression levels in the same types of cancer may due to the various detection methods, histological grade, or pathological stage. Second, the underlying mechanisms of miR-383 in the biological processes are complex and variable. We summarized the target genes and signaling pathways that were regulated by miR-383 in various tumors. However, many studies reported that the expression of miR-383 was markedly changed during tumorigenesis, while they did not explore the target genes. Therefore, other target genes and signal pathways of miR-383 should be revealed for further investigation. Third, up to now, most studies indicate that miR-383 functions as a tumor suppressor because it represses cell proliferation, decreases xenograft development, inhibits invasion and metastasis, promotes cell apoptosis, and sensitizes tumor cells to chemotherapy agents. However, it also has oncogenic functions in promoting proliferation, enhancing metastasis, and inducing tumorigenesis.

In conclusion, we summarized the aberrant expression of miR-383 in various human cancers, highlighting the functions of miR-383 in proliferation, development and differentiation, apoptosis, invasion and metastasis, as well as its roles in other human diseases. Additionally, we also reviewed the diverse target genes and signaling pathways regulated by miR-383 in cancer. This review provides some suggestions and evidence for further investigations and clinical applications. Although the relationship of miR-383 and tumorigenesis was explored, further investigations are required to explore its role in cancer biological behaviors. Future work will have help lead to strategies for cancer diagnosis and treatment.

## Figures and Tables

**Table 1:** miR-383 expression in various cancers and its target genes.

Cancer types	Expression	Target gene	Reference
Lung cancer	Decreased	*/, Wnt1, E2F7, EPAS1, CIP2A*	11,13-16
Glioma	Decreased	*PRDX3, IGF-1R, /, VEGF, CCND1*	17-21
Testicular embryonal carcinoma	Decreased	*PNUTS, IRF1*	22,23
Ovarian cancer	Decreased	*/, LDHA, TRIM27*	24-26
Colorectal cancer	Decreased	*PAX6, CREPT/RPRD1B, /*	27,28
Hepatocellular cancer	Decreased	*LDHA, APRIL, PHF8*	29-31
Medulloblastoma	Decreased	*/, FOXM1, PRDX3*	32-34
Pancreatic carcinoma	Decreased	*SOX11, GAB1, ROBO3*	35-37
Esophageal squamous cell carcinoma	Decreased	*SP1, 5S rRNA, MALAT1*	38-40
Breast cancer	Decreased	*LDHA, Gadd45g, PD-L1*	41-43
Gastric cancer	Decreased	*PP2A, HDAC9, ERBB4*	44-46
Cervical cancer	Decreased	*PARP2*	47
Colon cancer	Decreased	*APRIL*	48
Prostate cancer	Decreased	*CD44*	49
MALT lymphoma	Decreased	*ZEB2*	50
Pituitary adenoma	Decreased	*/*	51
Ependymoma	Decreased	*/*	52
Nasopharyngeal carcinoma	Decreased	*HMGA2*	53
Cholangiocarcinoma	Increased	*IRF1*	54
Epithelial ovarian cancer	Increased	*CASP2*	55
Canine malignant melanoma	Increased	ATR serine/threonine kinase*, CDK2*	56
Hepatocellular carcinoma	Increased	*/, EIF5A2*	57,58
Renal cell carcinoma	Increased	*DIO1*	59

“/” means no miR-383 targeted gene.

**Table 2:** Summarization of ceRNAs-miR-383 networks.

Cancer types or Diseases	LncRNAs or circRNAs	Expression	Reference
Esophageal squamous cell carcinoma	LncRNA-FGD5-AS1	Increased	38
Pancreatic carcinoma	lncRNA-TMPO-AS1	Increased	35
Lung cancer	lncRNA-TMPO-AS1	Increased	107
Glioma	lncRNA-TMPO-AS1	Increased	108
Glioma	LINC01614	Increased	109
Glioma	LINC00162	Increased	110
Oral Squamous cell carcinoma	RP11-284F21.9	Increased	111
Cervical cancer	LINC01128	Increased	112
Hepatocellular carcinoma	LncRNA-PTTG3P	Increased	113
Liver cancer	LncRNA-HULC	Increased	114
Prostate cancer	LncRNA-SNHG1	Increased	115
Breast cancer	LINC00096	Increased	116
Head and neck squamous carcinoma	lncRNA-HOXC-AS	Increased	53
Head and neck squamous carcinoma	LncRNA-MIR4435-2HG	Increased	117
Nephropathy	LncRNA-PTTG3P	Increased	118
Diabetic retinopathy	LINC00162	Increased	119
Diabetic retinopathy	lncRNA-LUADT1	Increased	120
Diabetic retinopathy	lncRNA-AK077216	Decreased	121
Recurrent pregnancy loss	LncRNA-MALAT1	Decreased	101
Spinal cord injury	lncRNA-CASC9	Decreased	122
Lung cancer	CircRNA-CCS	Increased	14
Colorectal cancer	CircRNA-0136666	Increased	106
Medulloblastoma	CircRNA-SKA3	Increased	33
Breast cancer	CircRNA-0001791	Increased	123

## Abbreviations

MiRNAs: microRNAs
UTR: Untranslated Region
UV: Ultraviolet radiation
EMT: Epithelial to mesenchymal transition
TNF-α: Tumor necrosis factor-α
TGF-β1: Transforming growth factor β 1
ATR: ATR serine/threonine kinase
LPS: lipopolysaccharide
ATM: ATM serine/threonine kinase
MALT lymphoma: Mucosa-associated lymphoid tissue lymphoma
ROS: Reactive Oxygen Species
CLL: Chronic lymphocytic leukemia
NSCLC: Non-Small-Cell Lung Cancer
DF: Dengue fever
UC: ulcerative colitis
CD: Crohn disease
GECs: Glioma-exposed endothelial cells
MSCs: Mesenchymal stem cells
LDHA: Lactate dehydrogenase A
VEGF: Vascular endothelial growth factor
FAK: Focal adhesion kinase
APRIL: A proliferating-inducing ligand
CDK2: Cyclin-dependent kinase 2
IRF1: Interferon regulatory factor 1
CCND1: Cyclin D1
IGF: Insulin like growth factor 1
IGF-1R: Insulin like growth factor 1 receptor
BCL-2: B cell lymphoma-2
SP1: Sp1 transcription factor
GDNF: Glial cell derived neurotrophic factor
PARP: Poly(ADP-ribose) polymerase
H2AX: H2AX variant histone
ZEB2: Zinc finger E-box binding homeobox 2
IRF1: Interferon regulatory factor 1
c-MYC: MYC proto-oncogene, bHLH transcription factor
MALAT1: Metastasis associated lung adenocarcinoma transcript 1
HMGA2: High mobility group AT-hook 2
PHF8: PHD finger protein 8
FOXM1: Forkhead box M1
SOX11: SRY-box transcription factor 11
GAB1: GRB2 associated binding protein 1
ROBO3: Roundabout guidance receptor 3
GADD45g: Growth arrest and DNA damage inducible gamma
PD-L1: CD274 molecule
PP2A: Protein phosphatase 2 phosphatase activator
HDAC9: Histone deacetylase 9
ERBB4: Erb-b2 receptor tyrosine kinase 4
PARP2: poly(ADP-ribose) polymerase 2
CD44: CD44 molecule
CASP2: Caspase 2
EIF5A2: Eukaryotic translation initiation factor 5A2
DIO1: Iodothyronine deiodinase 1
SGCZ: Sarcoglycan zeta
WNT1: Wnt family member 1
E2F7: E2F transcription factor 7
EPAS1: Endothelial PAS domain protein 1
CIP2A: Cellular inhibitor of PP2A
PRDX3: Peroxiredoxin 3
PNUTS: Protein phosphatase 1 regulatory subunit 10
RBMS1: RNA binding motif single stranded interacting protein 1
E2F1: E2F transcription factor 1
SF-1: Splicing factor 1
PPARγ: Peroxisome proliferator activated receptor gamma
PSD95: Discs large MAGUK scaffold protein 4
MMP: Matrix metalloproteinase
mTOR: Mechanistic target of rapamycin kinase
OCN: Bone gamma-carboxyglutamate protein
TRIM27: Tripartite motif containing 27
PAX6: Paired box 6
RPRD1B: Regulation of nuclear pre-mRNA domain containing 1B
